# Common folate gene variant, *MTHFR* C677T, is associated with brain structure in two independent cohorts of people with mild cognitive impairment^☆^

**DOI:** 10.1016/j.nicl.2012.09.012

**Published:** 2012-10-04

**Authors:** Priya Rajagopalan, Neda Jahanshad, Jason L. Stein, Xue Hua, Sarah K. Madsen, Omid Kohannim, Derrek P. Hibar, Arthur W. Toga, Clifford R. Jack, Andrew J. Saykin, Robert C. Green, Michael W. Weiner, Joshua C. Bis, Lewis H. Kuller, Mario Riverol, James T. Becker, Oscar L. Lopez, Paul M. Thompson, for the Alzheimer's Disease Neuroimaging Initiative (ADNI), the Cardiovascular Health Study (CHS)

**Affiliations:** a Laboratory of Neuro Imaging, Department of Neurology, UCLA School of Medicine, Los Angeles, CA, USA; b Medical Imaging Informatics Group, Department of Radiology, UCLA School of Medicine, Los Angeles, CA, USA; c Department of Radiology, Mayo Clinic, Rochester, Minnesota, USA; d Center for Neuroimaging, Department of Radiology and Imaging Science, Indiana University School of Medicine, Indianapolis, IN, USA; e Division of Genetics, Department of Medicine, Brigham and Women's Hospital and Harvard Medical School, Boston, MA, USA; f Department of Radiology, Medicine, and Psychiatry, University of California San Francisco, CA, USA; g Department of Veterans Affairs Medical Center, San Francisco, CA, USA; h Cardiovascular Health Research Unit and Department of Medicine, University of Washington, Seattle, Washington, USA; i Department of Epidemiology, University of Pittsburgh Graduate School of Public Health, Pittsburgh, PA, USA; j Department of Neurology, University of Pittsburgh School of Medicine, Pittsburgh, PA, USA; k Department of Neurology, Clínica Universidad de Navarra, Pamplona, Spain; l Department of Psychiatry, University of Pittsburgh School of Medicine, Pittsburgh, PA, USA; m Department of Psychology, University of Pittsburgh School of Medicine, Pittsburgh, PA, USA

## Abstract

A commonly carried C677T polymorphism in a folate-related gene, *MTHFR*, is associated with higher plasma homocysteine, a well-known mediator of neuronal damage and brain atrophy.

As homocysteine promotes brain atrophy, we set out to discover whether people carrying the C677T *MTHFR* polymorphism which increases homocysteine, might also show systematic differences in brain structure.

Using tensor-based morphometry, we tested this association in 359 elderly Caucasian subjects with mild cognitive impairment (MCI) (mean age: 75 ± 7.1 years) scanned with brain MRI and genotyped as part of Alzheimer's Disease Neuroimaging Initiative. We carried out a replication study in an independent, non-overlapping sample of 51 elderly Caucasian subjects with MCI (mean age: 76 ± 5.5 years), scanned with brain MRI and genotyped for *MTHFR*, as part of the Cardiovascular Health Study. At each voxel in the brain, we tested to see where regional volume differences were associated with carrying one or more *MTHFR* ‘T’ alleles.

In ADNI subjects, carriers of the *MTHFR* risk allele had detectable brain volume deficits, in the white matter, of up to 2–8% per risk T allele locally at baseline and showed accelerated brain atrophy of 0.5–1.5% per T allele at 1 year follow-up, after adjusting for age and sex. We replicated these brain volume deficits of up to 5–12% per *MTHFR* T allele in the independent cohort of CHS subjects.

As expected, the associations weakened after controlling for homocysteine levels, which the risk gene affects. The *MTHFR* risk variant may thus promote brain atrophy by elevating homocysteine levels.

This study aims to investigate the spatially detailed effects of this *MTHFR* polymorphism on brain structure in 3D, pointing to a causal pathway that may promote homocysteine-mediated brain atrophy in elderly people with MCI.

## Highlights

► Commonly carried *MTHFR* gene polymorphism affects plasma homocysteine levels. ► It also accelerates brain tissue loss in the elderly with mild cognitive impairment. ► We have mapped *MTHFR* brain structure associations in 3D in two independent cohorts. ► We suggest a causal pathway for homocysteine-mediated brain atrophy in the elderly.

## Introduction

As life expectancy increases, a rapidly increasing number of people worldwide are affected by degenerative brain disorders (Mattson et al., 2002). It is vital to identify factors that influence neurodegeneration to promote successful brain aging through genetic (germline gene therapy) and environmental (dietary and behavioral) modifications.

Several new factors have recently been discovered that promote or resist brain atrophy in the elderly. Among those that could in principle be influenced by pharmaceutical or even dietary interventions are factors in the folate/homocysteine pathway (Smith et al., 2010). Homocysteine is an amino acid measurable in the blood. Genetic variations explain 45–60% of the variance in plasma homocysteine levels in normal adults (Bathum et al., 2007; Siva et al., 2007).

A commonly carried genetic variant – a single nucleotide polymorphism (SNP) (C677T locus; rs1801133) in the well-known candidate gene methylenenetetrahydrofolate reductase (*MTHFR*) on chromosome 1 – explains a fair proportion (24–53%) of this genetic variance (Bathum et al., 2007). The C677T change in the *MTHFR* gene replaces cytosine with thymine at base position 677. Carriers of the T allele have a less active MTHFR enzyme and higher homocysteine levels, especially when their folate levels are low. This has been consistently shown in several genome-wide association studies (Hazra et al., 2009; Lange et al., 2010; Mälarstig et al., 2009; Pare et al., 2009; Raz et al., 2011; Tanaka et al., 2009). The *MTHFR* C677T ‘T’ allele has also been shown, albeit inconsistently, to be associated with white matter hyperintensities (de Lau et al., 2010; Hong et al., 2009; Kohara et al., 2003; Raz et al., 2011; Watfa et al., 2010).

This risk variant is widely prevalent in the United States: 40% and 11% of the non-Hispanic white population carry one or two risk alleles, respectively (Yu et al., 2010); the minor allele frequency is reported to be 31% in Western and Central Europeans (Altshuler et al., 2010).

Homocysteine is toxic to neurons via direct DNA damage and excitotoxicity, inducing apoptosis (Sachdev, 2005). High homocysteine levels have also been associated with brain atrophy in the elderly. Recently, we have shown that higher plasma homocysteine levels are associated with regional brain volume deficits in elderly Caucasian people, more specifically in people with mild cognitive impairment (MCI) (Rajagopalan et al., 2011).

**Fig. 1:**
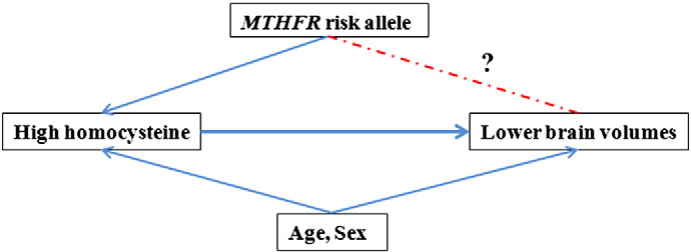
Established associations are shown (*solid lines*) based on the literature. We tested whether carriers of the *MTHFR* risk allele would show detectable differences on brain MRI (*dotted lines*).

If homocysteine is associated with brain atrophy in elderly people with mild cognitive impairment, then carriers of the *MTHFR* C677T risk polymorphism may show detectable differences in brain structure on MRI. By showing that brain structure is affected not just by homocysteine levels, but also by a commonly carried genetic variant that in turn elevates plasma homocysteine levels, a causal pathway to brain atrophy could begin to be elucidated — revealing brain systems affected, and the extent of their deficits in risk variant carriers (Fig. 1).

We hypothesized that we would find brain regions with structural deficits in subjects carrying the *MTHFR* risk allele. This study aims to investigate the spatially detailed effects of the homocysteine-associated *MTHFR* risk ‘T’ allele on brain structure in 3D.

## Materials and methods

### Subjects

Subjects were selected from two separate cohorts: the Alzheimer's Disease Neuroimaging Initiative (ADNI) (Table 1) and the Cardiovascular Health Study (CHS) (Table 2).

#### Alzheimer's Disease Neuroimaging Initiative (ADNI)

The ADNI was launched in 2004 as a 5-year public–private partnership to assess biomarkers of Alzheimer's disease (Mueller et al., 2005a,b). The first phase of ADNI (sometimes known as “ADNI1”) assessed 842 subjects in total — 200 with Alzheimer's disease (AD), 410 with MCI and 232 cognitively normal; mean age: 75·3 ± 6·8 years) at baseline who received a 1.5 Tesla brain MRI scan at one of 58 sites across North America and were genotyped as part of the study.

We considered the MCI group for our primary hypothesis, as MCI was the largest diagnostically homogeneous group in ADNI. In the MCI group, regional brain volumes showed strongest and most highly significant associations with homocysteine levels in our earlier work (Rajagopalan et al., 2011). In ADNI, almost the entire cohort was Caucasian; we therefore assessed only Caucasian (non-Hispanic) elderly subjects with MCI (n = 359) (Table 1) to avoid population stratification effects which can lead to spurious genetic associations (Lander and Schork, 1994). Of the 359 subjects, 171 had a history of hypertension, 8 had a history of stroke at baseline, 58 subjects had a history of coronary artery disease, 25 subjects had non-insulin dependent diabetes and 1 subject had insulin-dependent diabetes. We followed up 307 of these 359 MCI subjects after a 12-month interval, to test *MTHFR* polymorphism associations with the longitudinal rate of brain atrophy, estimated from the baseline and 1-year follow-up scans.

A second, purely exploratory, analysis was undertaken to test the associations of the *MTHFR* SNP in the AD and the cognitively normal control groups.

A post-hoc analysis was carried out using baseline and one-year bilateral cerebral white matter volumes (cc) based on the imaging data from the ADNI database processed by the UCSF team, who performed volumetric segmentations with the FreeSurfer image analysis suite (http://surfer.nmr.mgh.harvard.edu/). Volumes for 355 of the 359 Caucasian subjects at baseline and 303 of the 307 Caucasian subjects at one-year were reported to have passed quality control and were therefore used in the analysis.

The study was conducted according to the Good Clinical Practice guidelines, the Declaration of Helsinki, and US 21 CFR Part 50 — Protection of Human Subjects, and Part 56 — Institutional Review Boards. All participants gave written informed consent. Inclusion and exclusion criteria are detailed in the ADNI protocol (Mueller et al., 2005a). All data are publicly available (http://www.loni.ucla.edu/ADNI/).

#### The Cardiovascular Health Study (CHS)

We opted to test the *MTHFR* SNP in CHS subjects diagnosed with MCI, to replicate the associations found in the ADNI MCI group. CHS is an independently assessed cohort, and it was also analyzed here to determine whether the findings from ADNI might generalize to a larger population.

The CHS-CS (Cognition Study) was designed to determine the incidence of dementia and mild cognitive impairment (MCI) in a large cohort of subjects first assessed in 1998 — subjects were recruited from a Medicare database and were eligible if they were over age 65, ambulatory, and non-institutionalized. In 1998–1999, a subgroup of subjects had been identified as normal or MCI in 1991–1994. By 1999, 456 of these subjects had completed neurological and neuropsychological examinations and were scanned with 3-dimensional volumetric brain MRI.

Details of the CHS design have been described previously (Fried et al., 1991; Tell et al., 1993). T1-weighted brain MRI scans from 1998 to 1999 were analyzed from 97 elderly subjects diagnosed with MCI in the Cardiovascular Health Study (CHS) (mean age: 76.5 ± 5.5 years). As for ADNI study, we considered only a subset of 83 MCI Caucasian subjects to reduce population stratification effects. Of those 83 subjects, 51 had been genotyped for the *MTHFR* SNP. 15 of these subjects had data on homocysteine levels in the blood, 50 had data on folate levels, and 47 had been genotyped for *ApoE4* status (Table 2).

### MRI acquisition, calibration, and correction

#### ADNI baseline

Subjects were scanned at multiple ADNI sites according to a standardized protocol developed after a major effort to evaluate 3D T1-weighted sequences for morphometric analyses (Jack et al., 2008; Leow et al., 2006). See Supplementary materials for a detailed description.

#### 12-months follow-up

307 Caucasian subjects with MCI were scanned and analyzed at baseline (0 months), and at around one-year after their baseline scans. The follow-up slightly varied among the 307 subjects; 10 months for 1 subject, 12 months for 59 subjects, 13 months for 176 subjects, 14 months for 52 subjects, 15 months for 12 subjects and 16 months for 7 subjects. In these 307 subjects, we tested the associations of the *MTHFR* SNP with regional rate of brain atrophy over time computed across the 1-year interval. Rates of brain atrophy were computed with an inverse-consistent method, described previously (Leow et al., 2006).

#### CHS

All CHS MRI data used in this study were acquired at the University of Pittsburgh Medical Center (UPMC) Magnetic Resonance Research Center using a 1.5 T GE Signa scanner (GE Medical Systems, Milwaukee, Wisconsin, LX Version). A 3D volumetric spoiled gradient recalled acquisition (SPGR) sequence was obtained covering the whole brain (TE/TR = 5/25 ms, flip angle = 40°, number of excitations (NEX) = 1, slice thickness = 1.5 mm/0 mm interslice gap). Images were acquired with an in-plane acquisition matrix of 256 × 256 × 124 image elements, 250 × 250 mm field of view, and an in-plane voxel size of 0.98 mm^3^.

### Tensor-based morphometry (TBM) and 3D Jacobian maps in ADNI and CHS

For each cohort, T1-weighted structural brain MRI scans were analyzed using a standard protocol (Hua et al., 2008b) and an average brain template was constructed using brain MRI scans from 40 cognitively healthy elderly individuals to enable automated image registration, reduce statistical bias and to make it easier to detect statistically significant effects (Hua et al., 2008a,b). The two customized, study specific, minimal deformation templates (MDT) were created from the baseline MRI scans of 40 cognitively healthy elderly subjects for ADNI and CHS cohorts respectively. The MDTs therefore represent the healthy population average for each cohort respectively.

Non-linear registration was carried out to the study-specific template for each cohort to share a common coordinate system. For each subject, the local expansion factor of the 3D elastic warping transform, calculated as the determinant of the Jacobian matrix of the deformation, was plotted (Leow et al., 2005) to show relative volume differences between each individual and the common template, and reveal areas of structural volume deficits, or expansions, relative to the healthy population average.

TBM was also applied to the longitudinal ADNI dataset by using a nonlinear registration algorithm to match 3D baseline structural MR images with follow-up images acquired 12 months later as explained in our earlier work (Leow et al., 2009).

### DNA isolation and genotyping

#### ADNI

For the ADNI cohort, DNA extracted from peripheral B lymphocytes was analyzed on Human610- Quad BeadChip (Illumina). See Supplementary materials for a detailed description.

#### CHS

For the CHS cohort, DNA extracted from peripheral leukocytes was used to determine *MTHFR* C677T genotype, as previously described (Frosst et al., 1995; Longstreth et al., 2004).

### Testing associations of the MTHFR risk allele with brain structure

To test our primary hypothesis, associations between *MTHFR* genotype and regional brain structure were examined in the following groups — 1) ADNI subjects diagnosed with MCI at baseline, 2) ADNI subjects diagnosed with MCI 1 year after their baseline scan, and 3) CHS subjects diagnosed with MCI at baseline (for the replication study).

A secondary exploratory analysis was carried out to test for any associations of the *MTHFR* variant with brain structure in the ADNI subjects diagnosed with AD (n = 173) and in normal healthy controls (n = 206), individually, at baseline.

In each group of subjects, a multiple regression was carried out at each voxel, to test for the additive effect of carrying *MTHFR* risk alleles, controlling for age and sex, as in Eq. (1):

(1) $$y=\beta_{0}+\beta_{\mathrm{MTHFR}}MTHFR+\beta_{Ag\text{e}}Age+\beta_{\mathrm{Sex}}Sex+\varepsilon$$

Here the dependent variable, *y*, is a vector representing the determinant of the Jacobian matrix of deformation (which represents brain tissue deficit or excess relative to the average brain template) at a particular voxel. The independent measure, *MTHFR*, is a vector whose components are the integers 0, 1 or 2 across subjects. *Age* and *Sex* are vectors representing the age and sex of each subject, and ε is an error term. The regression model was fitted at each voxel to localize the evidence of association between the *MTHFR* risk allele and structural differences across the entire brain after adjusting for age and sex.

Homocysteine and *ApoE4* genotype were also independently controlled for in the regressions to rule out any confounding effect. The same regression analysis was repeated for the CHS MCI group of subjects. As folate levels in the blood were available for the CHS cohort unlike for ADNI, the regression model was fitted at each voxel across the brain, with and without adjusting for homocysteine, folate, and *ApoE4* levels, in addition to age and sex.

A post-hoc analysis regressing bilateral cerebral white matter volumes (cc) against *MTHFR* risk alleles was also carried out in ADNI subjects, after adjusting for age and sex, using the Stata software (StataCorp, 2011, College Station, Texas).

### Correcting for multiple statistical comparisons

Significance maps for the above regressions were corrected for multiple comparisons across voxels in the brain using the standard false discovery rate (FDR) method (Benjamini and Hochberg, 1995). We used a standard FDR correction for multiple statistical comparisons across all voxels in the cerebral cortex region excluding the brain stem, at the conventionally accepted level of *q* = 0.05. This means that only 5% of the voxels shown in the thresholded statistical maps are expected to be false positives. The critical *p-*value is reported, which represents the highest *p*-value threshold for which only 5% of the surviving voxels are expected to be false positives. Note that the critical *p*-value threshold for the map is generally larger when standardized effect sizes are larger.

## Results

The genotype groups (T/T, C/T, and C/C) did not differ in any demographic or clinical characteristics, for the ADNI (Table 1) and CHS (Table 2) cohorts, respectively. 14.1% subjects were homozygous and 43.4% were heterozygous for the *MTHFR* risk ‘T’ allele in the full ADNI cohort. In the ADNI MCI cohort, 14.8% subjects were homozygous and 43.7% heterozygous carriers of the risk T allele. In the CHS subjects diagnosed with MCI, 11.8% subjects were homozygous and 56.8% heterozygous carriers of the *MTHFR* risk T allele.

The three main findings from this study include significant associations of the *MTHFR* risk ‘T’ allele with (i) cross-sectional brain volume deficits in the bilateral periventricular fronto-parietal white matter in ADNI MCI subjects at baseline, (ii) longitudinal annual brain atrophy in the periventricular parietal white matter in ADNI MCI subjects at 12 month follow-up, and (iii) cross-sectional posterior parieto-occipital white matter deficits in baseline CHS MCI subjects.

**Fig. 2:**
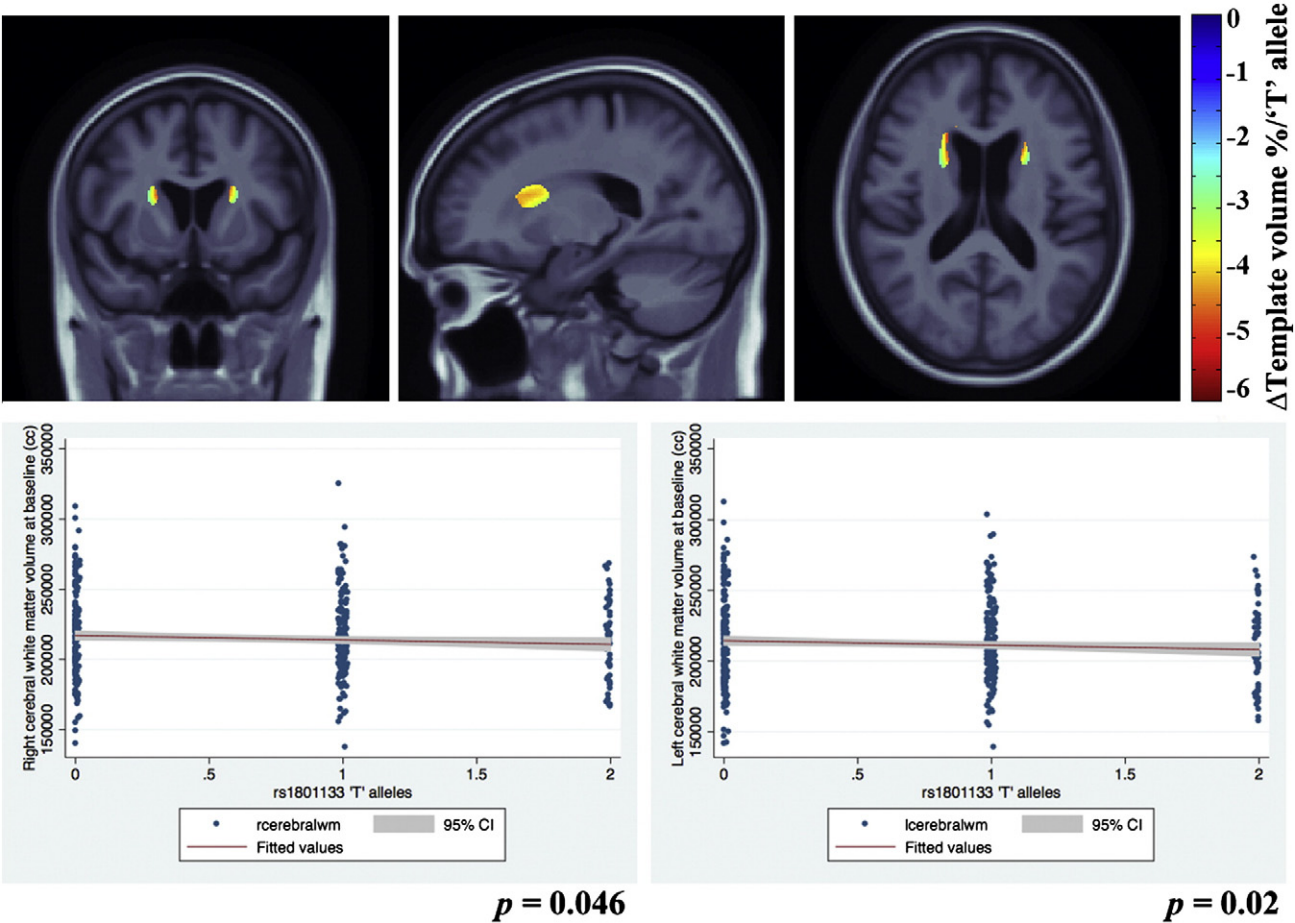
3D beta-coefficients maps show areas of significant volume deficits of up to 6% associated with a unit increase in carrying the *MTHFR* risk allele, with respect to the average template, in baseline ADNI subjects with mild cognitive impairment. Slices demonstrating the effects are depicted in the figure.

**Fig. 3:**
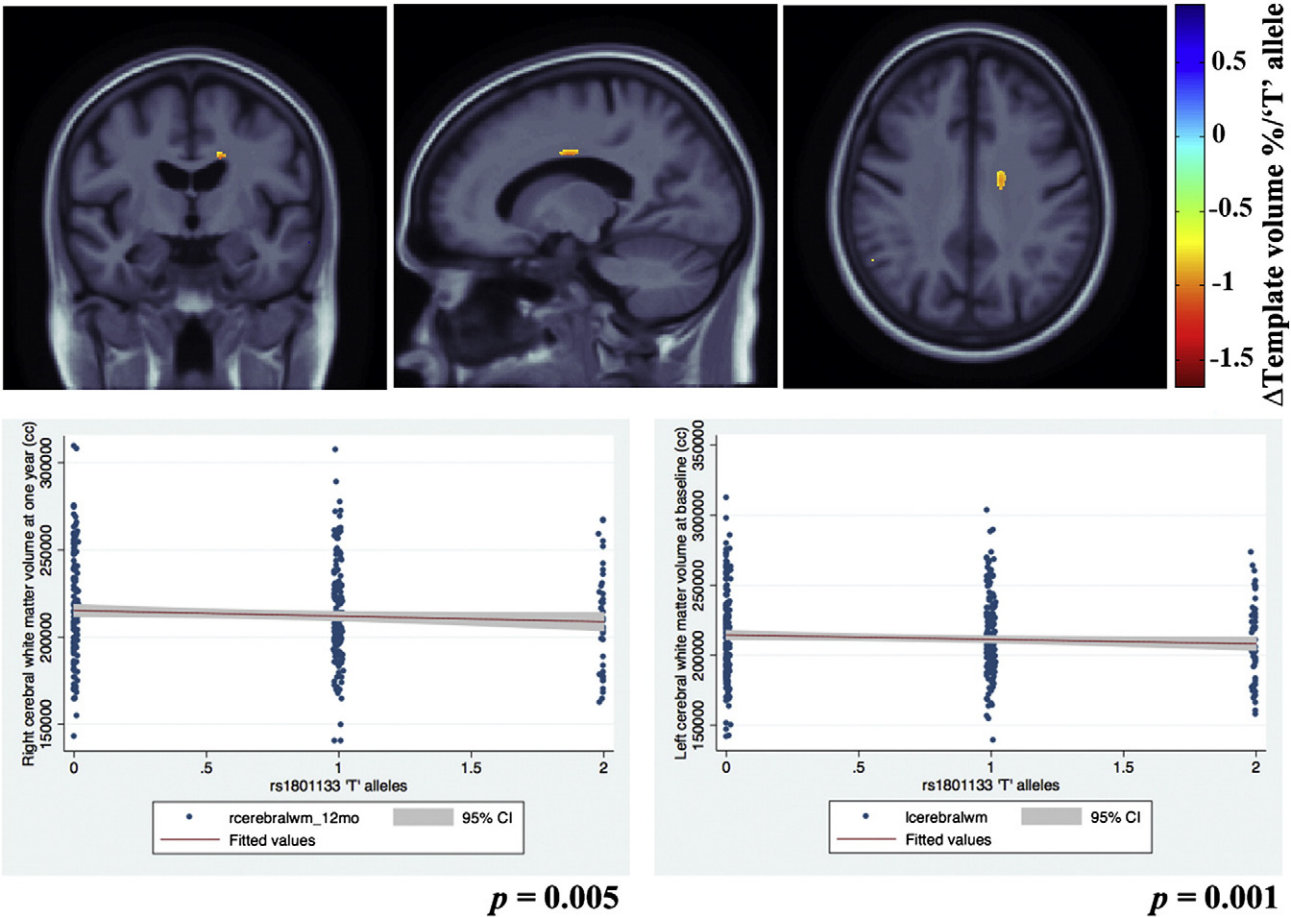
3D beta-coefficient maps show areas with significantly accelerated brain atrophy of up to 1.5% at 12 months follow-up, associated with a unit increase in carrying the *MTHFR* risk allele, in the same ADNI subjects with mild cognitive impairment.

Regression beta coefficient maps corrected for FDR are shown in Figs. 2 and 3 (*upper panel*) and 4, representing an estimate of the average percent brain tissue change for each unit change in the covariate studied. Images are in radiological convention (left side of the brain is shown on the right) and are displayed over the MDT. Warmer colors (*orange and red*) represent regions where each unit increase in the number of risk alleles is associated with a reduction in brain tissue. There are small regions of cooler colors (*blue*) in the CSF regions in the maps possibly due to partial volume effects (Hua et al., 2008a).

Graphs for the post-hoc analysis depicting the associations between bilateral cerebral white matter volumes and *MTHFR* risk allele in ADNI subjects, are shown in Figs. 2 and 3 (*lower panel*).

### *MTHFR* and ADNI baseline (cross-sectional) brain volumes

The additive effect of the risk ‘T’ allele was associated with significant regional brain volume deficits in white matter brain regions in the MCI group. Here the structural differences in the brain were significantly related to carrying the risk allele of the *MTHFR* gene after adjusting for age and sex (FDR critical *p*-value = 0.00043, controlling the standard FDR at the 5% level). In the significantly associated regions, average brain volume deficits of up to 6% per risk-conferring ‘T’ allele were found, bilaterally in the periventricular fronto-parietal white matter, relative to the MDT. Fig. 2 (*upper panel*) shows beta maps representing *MTHFR* associations after adjusting for age and sex and corrected for FDR.

To rule out spurious associations due to *ApoE4*, we also controlled for the presence of *ApoE4* alleles, even after which the *MTHFR* risk allele dose continued to show significant associations (FDR critical *p*-value = 0.0004 at *q* = 0.05). We also found no significant interactions between *ApoE4* and *MTHFR* risk alleles on brain volumes. Further, the *ApoE4* alleles are known to be associated with atrophy in bilateral temporal regions and expansion in the occipital horn of the lateral ventricle as shown in earlier work in ADNI (Hua et al., 2008a) and not in the white matter regions found to be associated with the *MTHFR* risk allele.

When controlling for homocysteine levels, the association was found to be significant (FDR critical *p*-value = 0.00038 at *q* = 0.05) but this effect was based on only a few scattered voxels and is not shown here.

In a post-hoc analysis with cerebral white matter volumes at baseline, we found *MTHFR* risk T allele to be significantly associated with left (*p* = 0.02) and right (*p* = 0.046) cerebral white matter volumes (Fig. 2 (*lower panel*)).

#### White matter hyperintensities and MTHFR risk allele

We did not find any significant associations of *MTHFR* risk allele with white matter hyperintensities (*p*-value = 0.54).

#### Secondary analyses in AD and control groups in ADNI

*MTHFR* polymorphism effects were not detectable in ADNI's small diagnostic subgroups of subjects with AD (n = 173) and the normal healthy controls (n = 206). Per ADNI's design, each of the non-MCI groups was considerably smaller than the MCI group (n = 359); ADNI deliberately scanned around twice as many MCI subjects as AD patients and controls. In our prior work (Rajagopalan et al., 2011), we found homocysteine effects were associated with brain volumes in MCI (n = 356 subjects) but not in the considerably smaller subgroups of AD patients (n = 173) and normals (n = 203). In the case of homocysteine effects, we attributed it to the smaller sample sizes, as it is somewhat unlikely that a gene effect is operating in MCI but not in people who are less or more ill. Here, in much the same way as the homocysteine effect, the *MTHFR* gene effect was picked up in the ADNI MCI group only – the largest subgroup – but not the two smaller subgroups (AD and controls from ADNI). Although it is tempting to think that sample sizes were too small to detect the effect reliably, there was a striking replication in the much smaller CHS cohort of just 51 subjects. Two interpretations are plausible: first is that there really is an effect in the CHS controls but not ADNI, or that the effect is stronger in CHS than in ADNI, or second that the effect is truly there all the time, but some methodological issue made it less easy to pick up in the ADNI controls. As noted in our prior studies of obesity in CHS and ADNI (Ho et al., 2010), the CHS cohort tends to have a higher prevalence of vascular disease and *ApoE4* genotypes than ADNI, and there are demographic differences too. Perhaps the most nuanced explanation is that the gene effect could be operating in some cohorts but not in others, and in some cohorts its effects may change with time; these second-order effects will be explorable in future in much larger sample sizes (e.g., via the Enigma Consortium; http://enigma.loni.ucla.edu). We also note that in the cohorts where the gene effect was detected, the pattern of findings for the genetic association is similar to the homocysteine associations with brain volumes in our earlier work (Rajagopalan et al., 2011) and probably also explains the effects of *MTHFR* risk allele on brain volumes in these subjects.

### MTHFR and the (longitudinal) rate of brain atrophy (at 1-year follow-up)

We also assessed brain structure in the 307 Caucasian ADNI MCI subjects after 12 months, and found similar statistically significant associations of the *MTHFR* C677T risk allele with the annual rate of brain volume atrophy, after adjusting for age and sex (FDR critical *p*-value = 0.00043 controlled for the standard FDR at the 5% level). The risk allele of the *MTHFR* SNP was associated with a significantly greater rate of progressive atrophy of up to 1.5% per year per ‘T’ risk allele, in the right side periventricular parietal white matter, relative to the MDT. Fig. 3 (*upper panel*) shows beta maps for regional *MTHFR* associations after adjusting for age and sex and corrected for FDR.

In a post-hoc analysis with cerebral white matter volumes at 12 months, as expected, we found *MTHFR* risk T allele to be significantly associated with left (*p* = 0.001) and right (*p* = 0.005) cerebral white matter volumes (Fig. 3 (*lower panel*)).

We controlled for the presence of *ApoE4* alleles, and carriers of the *MTHFR* risk alleles still showed accelerated brain atrophy of up to 1.5% (FDR critical *p*-value = 0.0004 at *q* = 0.05) in the same regions. There were no significant interactions between *ApoE4* and *MTHFR* risk alleles on brain volumes.

As expected from the known mechanism of the gene (it increases homocysteine levels), the associations weakened considerably when controlled for homocysteine. This is exactly as would be expected, if the allele effect may be partially mediated by homocysteine levels.

### Replication study: CHS MCI subjects — *MTHFR* and baseline brain volumes

**Fig. 4:**
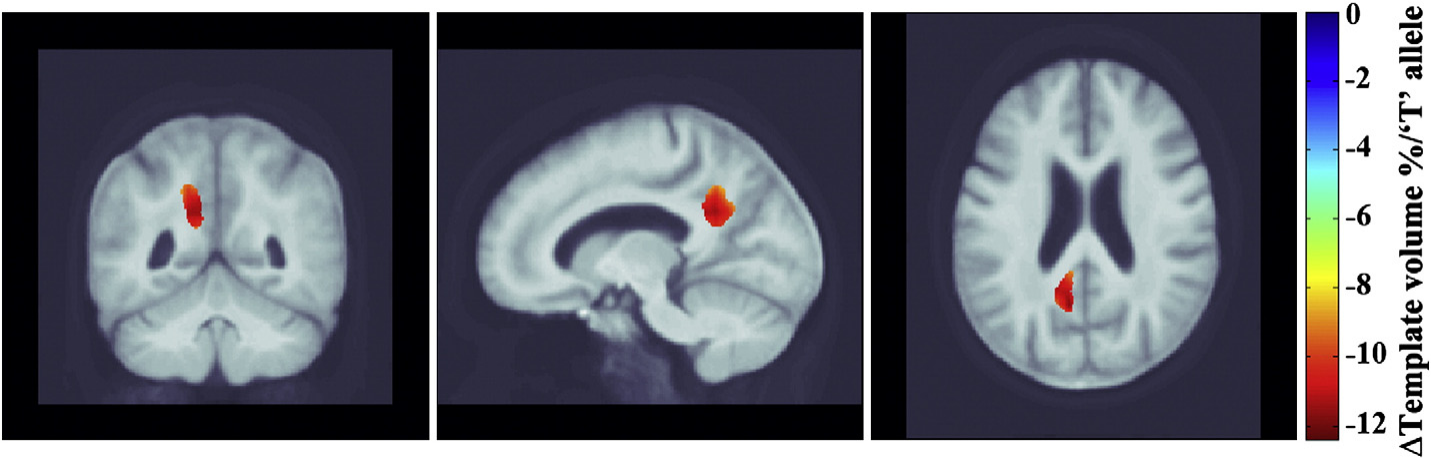
3D beta-coefficients maps show areas of significant volumes deficits of up to 12% associated with a unit increase in carrying the *MTHFR* risk allele, with respect to the average template, in baseline CHS subjects with mild cognitive impairment.

To replicate our SNP association finding, we carried out a similar analysis in the MCI subjects from the CHS dataset. We found significant associations between the *MTHFR* risk alleles and brain structure after controlling for age and sex (FDR critical *p*-value = 0.00043 controlled the standard FDR at the 5% level). Fig. 4 shows beta maps depicting *MTHFR* associations after adjusting for age and sex and corrected for FDR. This homocysteine-associated risk allele is associated with posterior parieto-occipital white matter deficits of up to 12% per risk T allele, relative to the MDT in this group.

The associations of *MTHFR* risk allele with brain volumes remained significant after adjusting for *ApoE4* genotype (n = 47) in addition to age and sex (FDR critical *p*-value = 0.0004 controlled the standard FDR at the 5% level).

The associations of *MTHFR* risk allele with brain volumes remained significant after controlling for folate levels in the blood (n = 50) in addition to controlling for age and sex (FDR critical *p*-value = 0.0004 controlled the standard FDR at the 5% level). As expected, the associations were no longer significant when controlled for plasma homocysteine levels, which the gene also affects.

## Discussion

This is a novel study aimed to discover characteristic voxel-wise patterns of brain atrophy associated with carrying the commonly prevalent *MTHFR* C677T gene variant, in 3D.

We found that a very commonly carried variant in the *MTHFR* gene, which is associated with high homocysteine levels in the blood, is significantly associated with brain structure variation, in particular with lower regional brain volumes, in subjects with MCI in both the ADNI and CHS cohorts. As these cohorts were independently collected and assessed, the results strongly corroborate our findings.

Consistent with our hypotheses, our main findings were:

(a) carrying *MTHFR* risk alleles was associated with having smaller brain volumes in MCI subjects from the ADNI cohort (Fig. 2); (b) carrying *MTHFR* risk alleles was associated with an accelerated rate of regional brain atrophy (Fig. 3) in the same MCI subjects from the ADNI cohort followed up after 12 months; (c) the *MTHFR* associations with lower regional brain volumes were replicated in an independent set of subjects with mild cognitive impairment from the CHS cohort (Fig. 4); (d) the regions of associations were not influenced by carrying *ApoE4* alleles in either cohort; (e) folate levels were controlled for in the CHS study and did not affect the results, so the *MTHFR* SNP effect on brain volumes was not dependent on the folate levels; (f) plasma homocysteine levels possibly mediate the effect: adjusting for homocysteine, as expected, weakens the associations of the *MTHFR* risk alleles with brain volumes in both cohorts.

The affected periventricular fronto-parietal white matter (Figs. 2 and 3) in ADNI and parieto-occipital white matter brain regions in CHS (Fig. 4) were identified as associated with homocysteine levels in our prior work on ADNI MCI subjects (Rajagopalan et al., 2011). These are regions in which white matter hyperintensities (associated with vascular infarcts) are most commonly detected in elderly individuals (Enzinger et al., 2006). However, the associations with white matter hyperintensities were not significant in our subjects. This is quite interesting in itself. It could either be that the WMH measure used in ADNI is fairly noisy — as it depends to some extent on thresholding the intensities of the scans to pick up what can sometimes be a very diffuse effect. Or it may be that the very subtle interval changes in brain volume are more precisely quantified by methods such as TBM, and the precision in the measures makes it easier to pick up a correlation with other pertinent biomarkers. There is some evidence in the latter direction, as drug trials using WMH volumes as an outcome measure sometimes need very large sample sizes, at least relative to measures of volumetric atrophy, which are easier to measure precisely.

It is somewhat disappointing that the brain regions showing associations with the *MTHFR* allele did not overlap in the ADNI and the CHS datasets after statistical thresholding, although the allele effects are likely more pervasive than the regions that survive thresholding. The CHS MCI cohort tended to show more posterior periventricular deficits when compared to the ADNI MCI cohort that showed in the fronto-parietal region, after adjusting for age and sex in the regression models. Also, the *MTHFR* risk allele associated brain volume deficits were somewhat stronger in the CHS (up to 12%; Fig. 4) than in ADNI (up to 6%; Fig. 2). These findings may reflect differences in the demographics of the two cohorts; the subtypes of MCI subjects in the ADNI (includes only amnestic MCI) is somewhat different from the CHS (includes probable and possible amnestic MCI). Effects of MCI subtypes may influence the strength and location of correlations between brain structure and the *MTHFR* risk allele. Also, as we used FDR to assess effects in the brain, we did not expect a strong congruence in the localization of effects, bearing in mind that the peaks of effect size in any one sample depend strongly on the noise and any unmodeled biological variation in the data. A similar situation was found in Kohannim et al. (2012), where we found significant, diffuse effects of the autism risk gene, *MACROD2*, on brain volumes in two different cohorts, but not in exactly the same locations in the brain. As such, we did not make a strong *a priori* hypothesis about the exact location of the effects, as any clusters in the statistical fields should not be considered the only places where biological effects are found, but simply evidence that there is a distributed effect on the brain. The overall significant associations between the *MTHFR* allele and brain volumes, in ADNI and CHS subjects alike, probably provide evidence that there is a distributed effect of the *MTHFR* allele on the brain; thereby providing support for considering replication of the *MTHFR* associations with brain volumes.

When controlling for the effects of the *ApoE4* allele, in addition to age and sex, there was no effect on the associations of the *MTHFR* risk allele and brain structure. *ApoE4* risk alleles are associated with temporal lobe volume deficits and ventricular expansion (Hua et al., 2008a; Schuff et al., 2009), but the profile of these deficits does not appear to interact with, or underlie, the associations of *MTHFR* risk allele. Therefore, *MTHFR* risk alleles are likely to exert their influence on brain structure independent of *ApoE4*.

Folate is essential for the stability and synthesis of myelin basic protein, which, in turn, is essential for white matter structure in the central nervous system. It acts as a coenzyme for a large number of metabolic reactions in the body and affects brain structure independent of plasma homocysteine levels. Also, the effects *MTHFR* variant on plasma homocysteine levels are pronounced when serum folate levels are low. Therefore, we adjusted for folate in the *MTHFR* SNP associations with brain structure in the CHS study but found that it did not affect the associations. In the ADNI study – unlike the CHS – folate levels were not measured in the subjects; so we could not test for *MTHFR* associations with brain structure, independent of folate levels in ADNI subjects. Folate supplementation is known to improve cognitive performance in the elderly with dementia and elevated plasma homocysteine (Nilsson et al., 2001). We were unable to look into folate supplements as we did not have the necessary data in this imaged cohort.

When we adjusted for homocysteine levels in ADNI and CHS, the correlations for the *MTHFR* C677T risk allele with brain volumes weakened, suggesting that the *MTHFR* effect on brain structure may in part be due to homocysteine elevation induced by the risk allele. The *MTHFR* risk allele may also directly affect brain structure independent of the homocysteine pathway. The *MTHFR* risk allele that is known to increase homocysteine levels has been shown, albeit inconsistently, to be associated with AD (Anello et al., 2004; Brunelli et al., 2001; Mansoori et al., 2011; Prince et al., 2001; Wang et al., 2005), vascular dementia (Chapman et al., 1998; Pollak et al., 2000), silent brain infarcts (Kohara et al., 2003) and white matter hyperintensities (Hong et al., 2009; Kohara et al., 2003). This, by itself, may increase the *MTHFR* variant associated risk for brain atrophy through various mechanisms including plaques and tangles (Vermeer et al., 2007), in addition to increased homocysteine levels.

Our finding of the *MTHFR* gene variant associations with brain atrophy may have implications for randomized controlled trials of medications aiming to lower homocysteine levels to resist brain degeneration. Some clinical trials target the folate pathway specifically, and advocate taking high doses of B vitamins as a preventative measure against further brain atrophy (Smith et al., 2010) and cognitive decline (Jager et al., 2011) in subjects with mild cognitive impairment. In such trials, and in epidemiological studies, it may help to genotype at this *MTHFR* SNP, as these genotypes may influence homocysteine levels and the observed pattern of atrophy. Further cross-sectional and prospective studies will be helpful in replicating these findings.

Our study was carried out in a large well-characterized cohort (ADNI n = 359; CHS n = 51) with high-quality standardized brain MRI, and well-validated computational methods to map patterns and rates of brain atrophy at a local level. We found significant associations of the *MTHFR* SNP with brain volumes in the subjects diagnosed with MCI in both ADNI and CHS studies, even though the replication sample was relatively small (n = 51).

The minor allele frequency for the *MTHFR* SNP varies moderately across different populations worldwide, and is reported to be 31% in Western and Central Europeans, 33% in US Chinese, 16% in US Gujarati Indians, 41% in US Mexicans, and 12% in African Americans (Altshuler et al., 2010). The subjects analyzed in the study were of Caucasian origin in both the cohorts to avoid spurious results due to population stratification (Lander and Schork, 1994). As a result, care must be exercised in generalizing our findings to ethnic groups with different allele frequencies and possibly different environmental influences.

Homocysteine levels in the CSF and plasma are highly correlated (*r* = 0.85) (Selley et al., 2002) but it is difficult to ascertain whether plasma homocysteine and folate levels correlate between blood and cerebrospinal fluid, especially when their values are not very high, as in the subjects studied here. Therefore in addition to elevated homocysteine, several other mechanisms such as dietary methionine intake, toxic habits, and pyridoxal-phosphate levels may contribute towards the effect of the *MTHFR* SNP on brain structure. Unfortunately, these measures were not available to us in the current study.

### Conclusions

These brain maps reveal that a commonly carried susceptibility allele for higher homocysteine is also associated with structural brain volume deficits and with accelerated rates of brain atrophy over time. As homocysteine is involved in this pathway and in the level of atrophy, the deficits may be resisted even in *MTHFR* gene risk allele carriers, at least in principle, via efforts to lower homocysteine levels. Healthy lifestyle changes such as increasing dietary folate and supplementation with B vitamins (Blaise et al., 2007; Smith et al., 2010) in carriers of the *MTHFR* risk variant may help slow the rate of atrophy, especially in elderly subjects with mild cognitive impairment.

## Figures and Tables

**Table 1:** Shown here are the ADNI MCI cohort's demographic and clinical characteristics, split by *MTHFR* (rs1801133) genotype.

Mean (standard error)	CC (homozygous non-risk genotype)	CT (heterozygous risk genotype)	TT (homozygous risk genotype)	ANOVA test results⁎
Sample size (n) (M — males, F — females)	149 (92 M, 57 F)	157 (103 M, 54 F)	53 (36 M, 17 F)	χ^2^_2_ = 0.8, *p* = 0.66
Age (years)	75 (0.5)	75 (0.5)	75 (0.9)	F_2, 356_ = 0.04, *p* = 0.96
BMIα (kg/m^2^)	25.8 (0.3)	26.5 (0.05)	25.7 (0.3)	F_2, 344_ = 1.26, *p* = 0.28
Systolic BPβ (mm Hg)	135.8 (1.1)	135.4 (1.2)	133.1 (1.9)	F_2, 256_ = 0.44, *p* = 0.64
Diastolic BPβ (mm Hg)	74.8 (0.6)	74.7 (0.7)	73.1 (1.0)	F_2, 356_ = 0.71, *p* = 0.49
White matter hyperintensity	0.8 (0.4)	0.9 (0.2)	1.0 (0.2)	F_2, 355_ = 0.18, *p* = 0.83
Homocysteineγ (μmol/L)	10.2 (0.2)	10.4 (0.2)	11.7 (0.05)	F_2, 352_ = 10.4, *p* < 0.0001⁎
MMSEδ	27.0 (0.2)	27.0 (0.2)	27.5 (0.3)	F_2, 356_ = 2.18, *p* = 0.11
Global CDRε	1.6 (0.2)	1.6 (0.1)	1.5 (0.2)	F_2, 356_ = 0.17, *p* = 0.85
*APOE*ε4 (0,1,2)	67, 68, 14	77, 56, 24	29, 19, 5	χ^2^_2_ = 2.24, *p* = 0.33

⁎ ANOVA tests were conducted to see if the mean clinical measure differed significantly across the genotype groups.

α Body mass index.

β Blood pressure.

γ Baseline morning fasting plasma levels.

δ Mini-mental status examination (maximum score: 30).

ε Clinical dementia rating.

**Table 2:** Shown here are the CHS MCI cohort's demographic and clinical characteristics, split by rs1801133 genotype.

Mean (standard error)	CC (homozygous non-risk genotype)	CT (heterozygous risk genotype)	TT (homozygous risk genotype)	ANOVA test results⁎
Sample size (n) (M — males, F — females)	16 (8 M, 8 F)	29 (11 M, 18 F)	6 (1 M, 5F)	χ^2^_2_ = 1.0, *p* = 0.37
Age (years)	74 (1.4)	77 (1.0)	73 (1.9)	F_2, 51_ = 1.9, *p* = 0.16
BMIα (kg/m^2^)	25.3 (1.4)	25.0 (1.0)	26.2 (1.1)	F_2, 44_ = 0.1, *p* = 0.89
Systolic BPβ (mm Hg)	128.7 (3.7)	138.3 (3.4)	133.3 (3.2)	F_2, 51_ = 1.5, *p* = 0.23
Diastolic BPβ (mm Hg)	65.4 (1.7)	69.3 (1.4)	67.1 (6.0)	F_2, 51_ = 0.9, *p* = 0.41
Homocysteineγ (μmol/L)	10.1 (0.2)	11.5 (0.2)	6.3ε	F_2, 15_ = 0.76, *p* = 0.49
Folateδ	534.7 (91.3)	537.1 (54.7)	372.7 (49.3)	F_2, 50_ = 0.6, *p* = 0.53

⁎ ANOVA and chi-squared tests were conducted to see if the mean clinical measure differed significantly across the genotype groups.

α Body mass index.

β Blood pressure.

γ Plasma homocysteine levels.

δ Folate levels.

ε Only 1 subject had data.
