# Supplementary material for: Common folate gene variant, MTHFR C677T, is associated with brain structure in two independent cohorts of people with mild cognitive impairment
Source: Neuroimage Clin. 2012 Oct 4;1(1):179–87. doi: 10.1016/j.nicl.2012.09.012 (PMC3757723; doi:10.1016/j.nicl.2012.09.012)
Supplement: Supplementary file 1 — Supplementary materials. [file mmc1.docx]

**Title (21 words)**

Common folate gene variant, *MTHFR* C677T, is associated with brain structure in two independent cohorts of people with mild cognitive impairment

**Abbreviated title (43 characters)**

*MTHFR* risk allele correlates with brain structure

Priya Rajagopalan^1^, Neda Jahanshad^1,2^, Jason L. Stein^1^, Xue Hua^1^, Sarah K. Madsen^1^, Omid Kohannim^1^, Derrek P. Hibar^1^, Arthur W. Toga^1^, Clifford R. Jack, Jr.^3^, Andrew J. Saykin^4^, Robert C. Green^5^, Michael W. Weiner^6,7^, Joshua C. Bis^8^, Lewis H. Kuller^9^, Mario Riverol^10,11^, James T. Becker^12^, Oscar L. Lopez^10^, Paul M. Thompson^1^ for the Alzheimer’s Disease Neuroimaging Initiative (ADNI)^13^, and the Cardiovascular Health Study (CHS)^14^.

^1^Laboratory of Neuro Imaging, Department of Neurology, UCLA School of Medicine, Los Angeles, CA, USA

^2^Medical Imaging Informatics Group, Department of Radiology, UCLA School of Medicine, Los Angeles, CA, USA

^3^Department of Radiology, Mayo Clinic, Rochester, Minnesota, USA

^4^Center for Neuroimaging, Department of Radiology and Imaging Science, Indiana University School of Medicine, Indianapolis, IN, USA

^5^Division of Genetics, Department of Medicine, Brigham and Women’s Hospital and Harvard Medical School, Boston, MA, USA

^6^Department of Radiology, Medicine, and Psychiatry, University of California San Francisco, CA, USA

^7^Department of Veterans Affairs Medical Center, San Francisco, CA, USA

^8^Cardiovascular Health Research Unit and Department of Epidemiology, University of Washington, Seattle, Washington, USA

^9^Department of Epidemiology, University of Pittsburgh School of Medicine, Pittsburgh, PA, USA

^10^Department of Neurology, University of Pittsburgh School of Medicine, Pittsburgh, PA, USA

^11^Department of Neurology, Clínica Universidad de Navarra, Pamplona, Spain

^12^Departments of Psychiatry and Psychology, University of Pittsburgh School of Medicine, Pittsburgh, PA, USA

^13^Investigators within ADNI contributed to the design and implementation of ADNI and/or provided data but not participate in analysis or writing of this report. For a complete listing of ADNI investigators, see <http://www.loni.ucla.edu/ADNI/Collaboration/ADNI_Manuscript_Citations.pdf>

^14^A full list of principal CHS investigators and institutions may be found at <http://www.chs-nhlbi.org/pi.htm>

**For submission to *NeuroImage – Clinical*: August 30, 2012**

**Corresponding Author**:

Paul Thompson PhD, Professor of Neurology and Psychiatry,

Imaging Genetics Center, Laboratory of Neuro Imaging, UCLA School of Medicine
635 Charles E. Young Drive South
Neuroscience Research Building, Suite 225
Los Angeles, CA 90095-7334
(310) 267-5069 Direct Line

[(310) 206-2101](tel:%28310%29%20206-2101) LONI Office
[(310) 206-5518](tel:%28310%29%20206-5518) Fax

Email: [thompson@loni.ucla.edu](mailto:thompson@loni.ucla.edu)

**Supplementary material**

***1. MRI acquisition, calibration, and correction***

*1.1 ADNI baseline*

High-resolution structural brain MRI scans were acquired using 1.5- and 3-Tesla (T) MRI scanners; however, we restricted our analysis to 1.5-T MRI scans, to avoid any confounding effect of scanner field strength on tissue volume quantification, and most subjects were scanned at 1.5-T ([Ho, et al., 2010](#_ENREF_1)). Images were calibrated with phantom-based geometric corrections to ensure consistency across scanners. Additional image corrections included: (i) correction of geometric distortions due to gradient nonlinearity, (ii) adjustment for image intensity inhomogeneity due to B_1_ field non-uniformity using calibration scans, (iii) reducing residual intensity homogeneity, and (iv) geometric scaling according to a phantom scan acquired for each subject to adjust for scanner- and session-specific calibration errors. Each incoming image file was quality checked for medical abnormalities and image quality. In addition, the ADNI scanning protocol was developed after a rigorous preparatory phase in which we and others made sure that the scan volumes were reproducible and stable across repeated scans, using the same volume quantification method as shown earlier ([Leow, et al., 2006](#_ENREF_3)).

***2. DNA isolation and genotyping***

*2.1 ADNI*

DNA was isolated from B lymphocyte cells taken from blood ([Neitzel, 1986](#_ENREF_4)) and extracted using standard procedures ([Lahiri, et al., 1992](#_ENREF_2)). Seven milliliters of EDTA blood were extracted using the QIAamp DNA Blood Maxi Kit (Qiagen). Samples were processed according to the manufacturer’s protocol. Genomic DNA samples were analyzed on the Human610- Quad BeadChip (Illumina) according to the manufacturer’s protocols (Infinium HD Assay; Super Protocol Guide; Rev. A, May 2008). Before initiating the assay, 50 ng of genomic DNA from each sample was examined qualitatively on a 1% Tris-acetate-EDTA agarose gel for visual signs of degradation. Any degraded DNA samples were excluded. Samples were quantitated in triplicate with PicoGreen reagent (Invitrogen) and diluted to 50 ng/μl in Tris-EDTA buffer (10 mM Tris, 1 mM EDTA, pH 8.0). Two hundred nanograms of DNA was then denatured, neutralized, and amplified for 22 h at 37 °C (termed the MSA1 plate). The MSA1 plate was then fragmented with FMS reagent (Illumina) at 37 °C for 1 h and then precipitated with 2-propanol and incubated at 4 °C for 30 min. The resulting blue precipitate was then resuspended in RA1 reagent (Illumina) at 48 °C for 1 h. The samples were then denatured (95 °C for 20 min) and immediately hybridized onto BeadChips at 48 °C for 20 h. BeadChips were then washed and subjected to single-base extension and staining. Finally, the BeadChips were coated with XC4 reagent (Illumina), dessicated, and imaged on the BeadArray Reader (Illumina).

**References**

Ho, A.J., Hua, X., Lee, S., Leow, A.D., Yanovsky, I., Gutman, B., Dinov, I.D., Leporé, N., Stein, J.L., Toga, A.W. 2010. Comparing 3 T and 1.5 T MRI for tracking Alzheimer's disease progression with tensor-based morphometry. Human brain mapping 31(4), 499.

Lahiri, D.K., Bye, S., Nurnberger, J.I. 1992. A non-organic and non-enzymatic extraction method gives higher yields of genomic DNA from whole-blood samples than do nine other methods tested. Journal of biochemical and biophysical methods 25(4), 193-205.

Leow, A.D., Klunder, A.D., Jack Jr, C.R., Toga, A.W., Dale, A.M., Bernstein, M.A., Britson, P.J., Gunter, J.L., Ward, C.P., Whitwell, J.L. 2006. Longitudinal stability of MRI for mapping brain change using tensor-based morphometry. NeuroImage 31(2), 627-40.

Neitzel, H. 1986. A routine method for the establishment of permanent growing lymphoblastoid cell lines. Human genetics 73(4), 320-6.
